# Bilateral Pulmonary Embolism in Patients Recovered From Asymptomatic COVID-19 Infection

**DOI:** 10.7759/cureus.13848

**Published:** 2021-03-12

**Authors:** Falmata Laouan Brem, Hammam Rasras, Noha El Ouafi, Zakaria Bazid

**Affiliations:** 1 Cardiology, Mohammed VI University Hospital, Mohammed I University, Oujda, MAR

**Keywords:** post-covid-19, asymptomatic disease, bilateral pulmonary embolism, thromboprophylaxis

## Abstract

Hypercoagulability state is common in patients with coronavirus disease 2019 (COVID-19), especially those requiring intensive care. Indeed, anticoagulation therapy is recommended for all hospitalized COVID-19-patients. Despite well-conducted thromboprophylaxis, so many patients have developed these life-threatening complications. However, these thrombotic events are not known to occur in asymptomatic COVID-19 patients or in those recovered from active severe acute respiratory syndrome coronavirus 2 infection. This case series of a bilateral pulmonary embolism that occurred in patients recovered from asymptomatic COVID-19 highlights the questions about extending thromboprophylaxis in ambulatory patients with COVID-19.

## Introduction

In December 2019, the emergence of the novel coronavirus (severe acute respiratory syndrome coronavirus 2 [SARS-CoV-2]) caused a global coronavirus disease 2019 (COVID-19) pandemic [1]. The initial presenting symptoms of the SARS-CoV-2 infection are mainly respiratory [2]. Nevertheless, non-respiratory manifestations are increasingly being recognized in COVID-19 [3]. Recently, a study reported approximately 40%-45% of persons developing asymptomatic COVID-19 with a possible association with subclinical lung abnormalities, as detected by computed tomography (CT) scans [4]. Thromboembolic events are common in COVID-19 patients. Among them, pulmonary embolism (PE) is the most frequent, especially in critically ill patients, despite anticoagulation therapy [5]. However, these events are not known to occur in asymptomatic COVID-19-patients or in those recovered from active SARS-CoV-2 infection. Here, we report three cases of a bilateral PE in patients recovered from asymptomatic COVID-19.

## Case presentation

This is a retrospective review of three bilateral pulmonary embolism cases in the recovery phase of asymptomatic COVID-19. They were seen at the University Hospital Center of Oujda between December 1, 2020 to January 12, 2021. We performed COVID-19 tests by serology and the SARS-CoV-2 reverse transcriptase-polymerase chain reaction (RT-PCR) test on nasopharyngeal swabs.

Case 1

**Figure 1:**
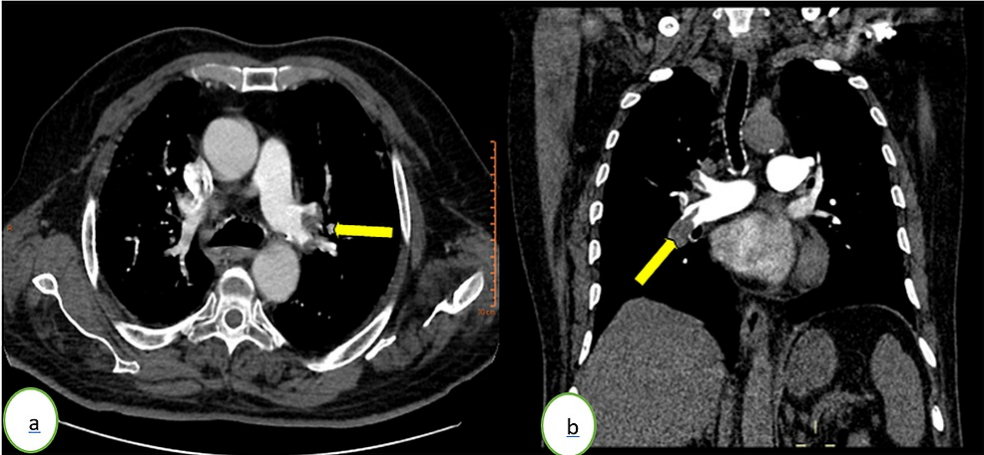
Computed tomography pulmonary angiography in (a) axial and (b) coronal windows showing a bilateral acute pulmonary embolism (yellow arrows) of the right pulmonary artery and a segmental branch of the left upper lobar branch

**Figure 2:**
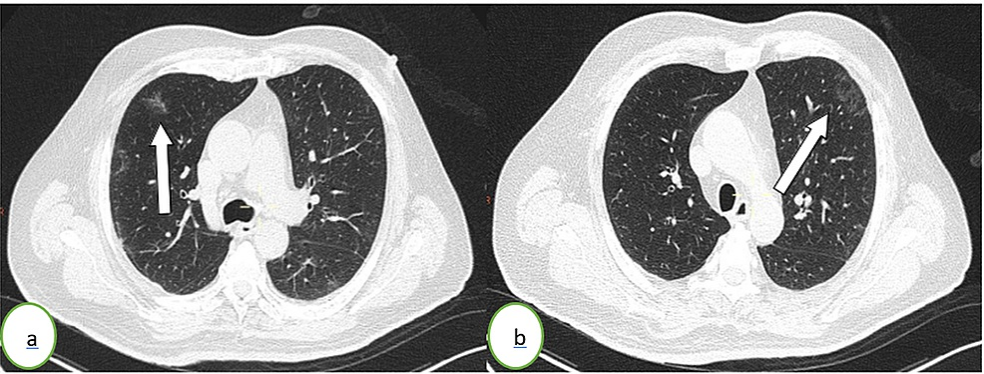
Thoracic CT scan in an axial (a, b) lung window showing subpleural ground-glass opacities (white arrows) in the left upper lung lobe The percentage of lung involvement is <10%.

A 68-year-old male with diabetes mellitus was admitted to the emergency department (ED) for sudden chest pain and dyspnea. Oxygen saturation was 84% at room air; the heart rate was 124 beats/min, and blood pressure was 100/70 mmHg. Electrocardiogram (EKG) showed a right bundle branch. The transthoracic echocardiography (TTE) showed a right ventricular (RV) dilatation with systolic dysfunction and paradoxical septum motion. The duplex ultrasound examination was normal. The CT pulmonary angiography (CTPA) showed a bilateral PE (Figure 1) with moderate signs of COVID-19 pneumonia (Figure 2). He received thrombolytic therapy with alteplase (PE was classified as intermediate-high risk with a simplified Pulmonary Embolism Severity Index [s-PESI] score of 3) and showed an excellent improvement. The patient had no symptoms suggestive of COVID-19 days before his admission. According to the COVID-19 pandemic, RT-PCR for COVID-19 was performed and showed a negative result, but the serology showed positive immunoglobulins M and G (IgM and IgG). Then he underwent a therapeutic dose of anticoagulation therapy with low-molecular-weight heparin. The patient was discharged seven days after his admission on rivaroxaban, with a close follow-up by his cardiologist.

Case 2

**Figure 3:**
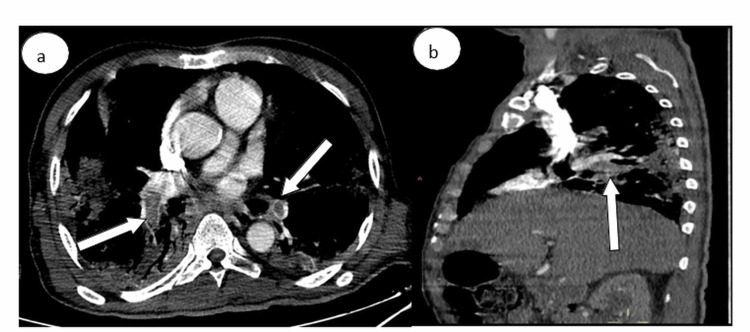
Computed tomography pulmonary angiography in (a) axial and (b) sagittal windows showing a bilateral pulmonary embolism (white arrows) of the two pulmonary arteries extended to the lobar branches

**Figure 4:**
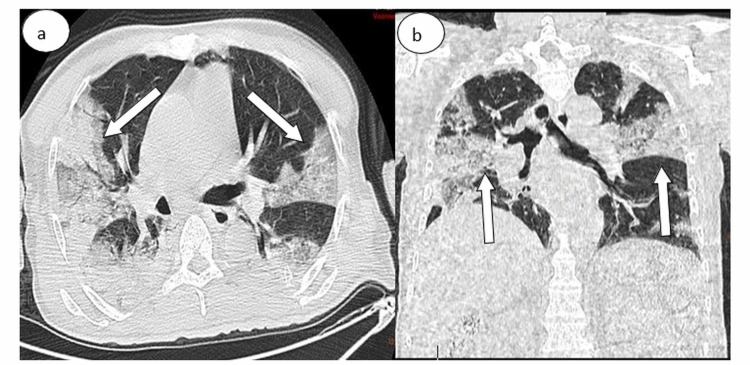
Thoracic CT scan in (a) axial and (b) coronal lung windows showing some ground-glass opacities (white arrows) associated with a bilateral massive consolidation The percentage of lung involvement is approximately 50%.

A 60-year-old male with diabetes mellitus was admitted to the ED for sudden dyspnea. Oxygen saturation was 85% at room air; the heart rate was 135 beats/min, and blood pressure was 120/85 mmHg. EKG was normal, and TTE showed a paradoxical septum motion with a good function of the right ventricle. The CTPA showed bilateral pulmonary embolism (Figure 3) with suggestive signs of SARS-CoV-2 pneumonia (Figure 4). According to the COVID-19 pandemic and suggestive radiological findings, we performed RT-PCR on a nasopharyngeal swab that showed a negative result. COVID-19 serology showed positive IgM and IgG. The patient had no symptoms suggestive of COVID-19 infection before his admission. The day after, the patient presented an acute respiratory failure with oxygen saturation at 45%. He was transferred to the intensive care unit and underwent a therapeutic dose of anticoagulation therapy with low-molecular-weight heparin. He deteriorated and deceased two days later.

Case 3

**Figure 5:**
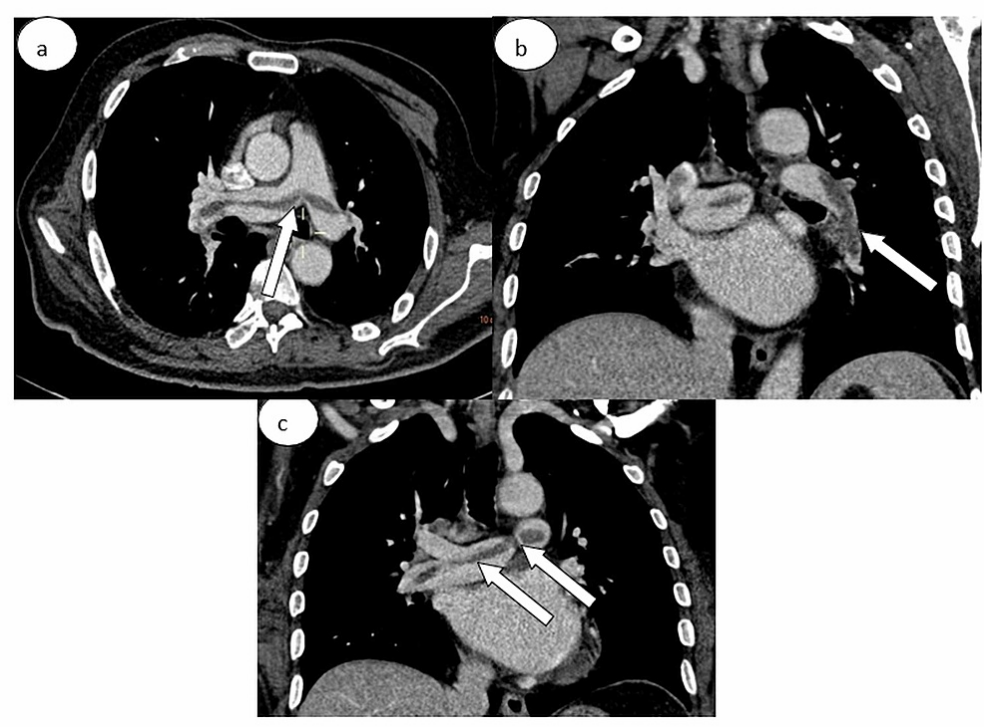
Computed tomography pulmonary angiography in (a) axial and (b, c) coronal windows showing the “railway sign” of acute pulmonary embolism (central filling defect surrounded by parallel lines of contrast) (white arrows) of the two pulmonary arteries, expanded to the left lower lobar branch

**Figure 6:**
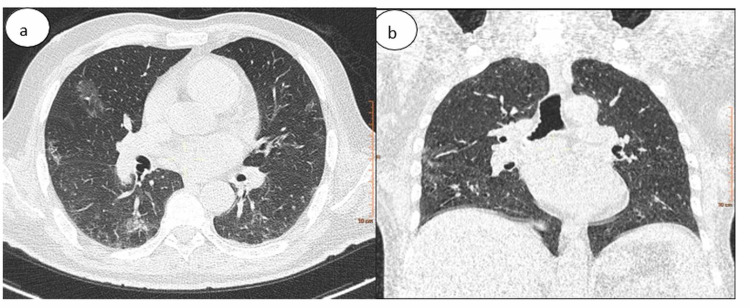
Chest CT in (a) axial and (b) coronal lung windows showing bilateral subpleural ground-glass opacities The percentage of lung involvement is approximately 25%.

An 85-year-old male presented to neurology consultation for an insidious paraparesis progression, which had begun 10 days earlier. Twenty-five days earlier, he had a positive COVID-19 RT-PCR performed after unprotected contact with his son, who was tested positive. He was not hospitalized and was discharged home. Two weeks later, his RT-PCR was negative. He had never developed symptoms suggestive of COVID-19 infection. As part of the paraparesis etiological assessment, a cerebral CT scan was performed, and the results were normal. Also, CT of the chest, abdomen, and pelvis was performed, which showed an insidious bilateral PE (Figure 5) with suggestive signs of COVID-19 pneumonia (Figure 6). The patient was hemodynamically stable, and oxygen saturation was 95% at room air. EKG and TTE were normal. The patient underwent a therapeutic dose of anticoagulation therapy with low-molecular-weight heparin, switched to rivaroxaban, and was followed up by his cardiologist.

## Discussion

To the best of the our knowledge, this is the first series of bilateral PEs in patients recovered from asymptomatic COVID-19 infection. Current data demonstrate that the hypercoagulability state is common in patients with COVID-19 disease. However, the pathogenesis remains unclear. Some authors suggested that the hypercoagulable state results from elevated coagulation blood factors such as factor VIII, D-dimer, fibrinogen, in addition to the dysfunction of the endothelial cells [5]. Besides, the blood flow stasis due to immobilization can occur in all hospitalized patients [6].

Indeed, PE is the most frequent thromboembolic complication during the COVID-19 disease, especially in critically ill patients [5]. Furthermore, this hypercoagulable state seems to persist even after the hospital discharge of patients with COVID-19, despite well-conducted thromboprophylaxis [7]. However, these events are not known to occur in asymptomatic COVID-19 patients or in those recovered from active SARS-CoV-2 infection. Therefore, the incidence of PE among ambulatory COVID-19 patients is not yet clearly defined. There have been a few reports of pulmonary thromboembolic events in recovered patients with COVID-19 [7-9].

Although there is an increased risk of venous thromboembolism (VTE) in patients with COVID-19, current guidelines do not recommend thromboprophylaxis in outpatients with COVID-19 [10]. In our cases, patients did not report reductions in mobility. Therefore, it is better to suggest that there may be a potential risk of VTE even in COVID-19 outpatients and not only critically ill patients. Other reports support our findings [11-12]. Although the prophylactic dose is not recommended in non-hospitalized patients [10], we suggest that outpatients with COVID-19, especially those with reduced mobility, should be regularly contacted and well informed about the possible risk of VTE. Our patients were asymptomatic. There have been reported cases of PE in asymptomatic COVID-19 patients [13-14] suggesting that in patients with unexplained VTE and no venous thrombosis risk factors, the SARS-CoV-2 infection should be screened even after recovery since it could be fatal [15].

## Conclusions

Although current guidelines do not recommend using thromboprophylaxis in COVID-19 outpatients, these cases of asymptomatic outpatients with COVID-19 developing PE illustrate the possible higher risk of VTE in ambulatory patients with COVID-19 compared to other medical illness. However, randomized controlled trials are needed to assess primary thromboprophylaxis's net benefit in outpatients with COVID-19.
